# Remarkable Stability of Myelinating Oligodendrocytes in Mice

**DOI:** 10.1016/j.celrep.2017.09.050

**Published:** 2017-10-10

**Authors:** Richa B. Tripathi, Martyna Jackiewicz, Ian A. McKenzie, Eleni Kougioumtzidou, Matthew Grist, William D. Richardson

**Affiliations:** 1 Wolfson Institute for Biomedical Research, University College London, Gower Street, London WC1E 6BT, UK

**Keywords:** myelin, internode, cell survival, cell turnover, transgenic mouse, Opalin-CreER, corpus callosum, cerebral cortex, spinal cord, optic nerve

## Abstract

New myelin-forming oligodendrocytes (OLs) are generated in the mouse central nervous system during adulthood. These adult-born OLs might augment the existing population, contributing to neural plasticity, or else replace OLs that die in use (turnover). To distinguish between these alternatives, we induced genetic labeling of mature myelinating OLs in young adult mice and tracked their subsequent survival. OL survival rates were region dependent, being higher in corpus callosum (∼90% survival over 20 months) and motor cortex (∼70% survival) than in corticospinal tract or optic nerve (50%–60% survival). Survival rates over the first 8 months were 90%–100% in all regions except the optic nerve. In the corpus callosum, new OLs accumulate during young adulthood and are therefore likely to participate in adaptive myelination. We also found that the number of myelin internodes maintained by individual cortical OLs is stable for at least 8 months but declines ∼12% in the following year.

## Highlights

• Myelinating oligodendrocytes (OLs) continue to develop in the adult mouse CNS
• They might replace dying OLs or else contribute to new (adaptive) myelination
• We estimated OL lifetime by genetic fate-mapping in *Opalin–iCreER*^*T2*^*: Tau–mGFP* mice
• In most regions, >90% of OLs survive >8 months, so most new OLs are probably adaptive

## Graphical Abstract

**Figure:**
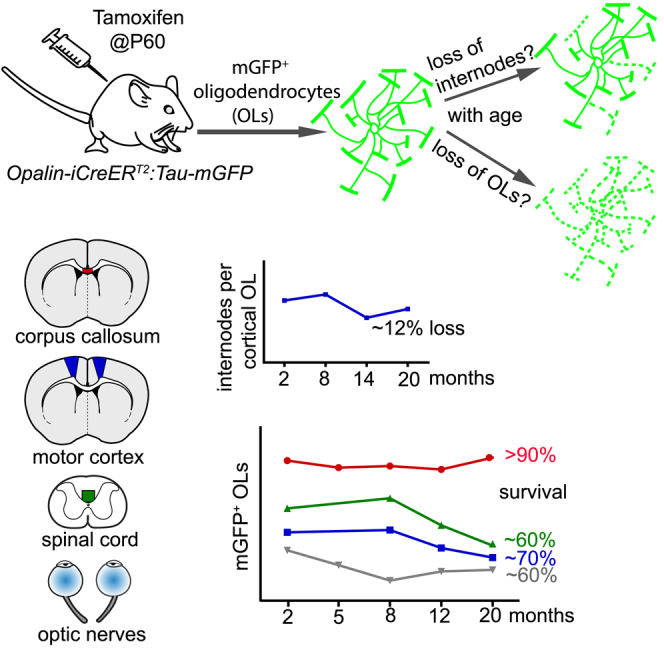

## Teaser

Tripathi et al. estimate the lifetime of myelinating oligodendrocytes (OLs) by fate-mapping in *Opalin-CreER*^*T2*^*: Tau-mGFP* mice. In the corpus callosum, >90% of OLs survived for >1.5 years and probably outlive the mouse. Therefore, adult-born OLs are not needed for myelin homeostasis but potentially contribute to experience-dependent, adaptive myelination.

## Introduction

Oligodendrocytes (OLs) myelinate axons in the CNS. In mice, most OLs are formed in the first ∼6 postnatal weeks, but they continue to be generated from OL precursors (OPs) for an extended period into adulthood. Several labs used transgenic mice that express tamoxifen-inducible Cre recombinase together with a Cre-dependent reporter to label OPs in young adult mice and follow their fates over time (Dimou et al., 2008, Rivers et al., 2008, Zhu et al., 2008, Guo et al., 2010, Kang et al., 2010). This revealed that OPs continue to generate myelin-forming OLs in several CNS regions for at least the first 8 months of life, though at a steadily decreasing rate (Psachoulia et al., 2009, Young et al., 2013). There is evidence that new OLs are still made in very small numbers even 1 year after birth (Xiao et al., 2016). This raises the question of why new OLs are needed in the adult mouse CNS.

Genetic blockade of new OL production in young adult mice prevented them from mastering a new motor skill (running at speed on a “complex wheel” with unevenly spaced rungs), implying that adult-born OLs are required for motor skill learning (McKenzie et al., 2014, Xiao et al., 2016). Consistent with this, wild-type mice that encountered the complex wheel for the first time transiently increased their production of new OLs and myelin in the motor cortex and subcortical white matter (McKenzie et al., 2014, Xiao et al., 2016). These findings are consistent with two conceptually different models for how new OLs and myelin participate in learning and memory processes: (1) preexisting myelinated circuits might need to be kept in a constant state of repair in order to be competent for learning, and new OLs are required for this maintenance function; or (2) learning might require that new circuits are brought into play, or existing circuits modified, through de novo myelination of previously unmyelinated or incompletely myelinated axons. To distinguish between these models, we need to know whether myelinating OLs have a limited survival lifetime and are continuously regenerated during adulthood, which would tend to favor the first model, or whether they are long-lived and accumulate throughout life, which would favor the second model.

To address this question, we estimated the survival rates of myelinating OLs in mice using a genetic fate-mapping approach. We generated a new line of *Opalin–iCreER*^*T2*^*: Tau–mGFP* transgenic mice, which express a membrane-bound form of GFP (mGFP) in mature myelinating OLs after tamoxifen-activated Cre recombination. This allowed us to induce labeling of myelinating OLs in adult mice and track their survival subsequently. We found that the great majority (∼90%) of myelinating OLs that were present in the corpus callosum at postnatal day 60 (P60) survived until at least 20 months of age. In the motor cortex, essentially all myelinating OLs survived during the first 8 months of life, and ∼70% survived for 20 months. There was no reduction in the number of myelin sheaths (internodes) synthesized by individual mGFP-labeled OLs in the motor cortex during at least the first 8 postnatal months. Therefore, new OLs that differentiate from OPs in the motor cortex and underlying white matter during early adulthood are not needed to replace OLs that die but rather add to the OL population, potentially modifying the existing circuitry (adaptive myelination). Consistent with this, we determined that the total number of mature OLs in the corpus callosum continued to increase up to ∼8 months of age.

In the spinal cord and optic nerve, 30%–50% of OLs that were present at P60 were lost over the following 18 months. Therefore, newly forming OLs in some regions of the adult CNS, especially after middle age, might be required for myelin homeostasis.

## Results

### Generation and Characterization of *Opalin-iCreER^T2^* Mice

Opalin (also known as Tmem10) is a transmembrane glycoprotein that is expressed in the cell bodies and processes of differentiated OLs (Golan et al., 2008, Kippert et al., 2008, Yoshikawa et al., 2008). It starts to be expressed in OLs after myelin sheath proteins such as myelin basic protein (MBP) and is not expressed in the peripheral nervous system or non-neural tissues. We generated a line of *Opalin–iCreER*^*T2*^ bacterial artificial chromosome (BAC) transgenic mice and crossed this with the *Rosa26–eYFP* reporter (*Rosa–YFP*; Srinivas et al., 2001). We injected tamoxifen (55 mg/kg body weight) into double-transgenic offspring starting on P60, and 10 days later (P60+10), we observed yellow fluorescent protein (YFP) immunolabeling of mature CC1^+^ OLs but not PDGFRA^+^ OPs, GFAP^+^ astrocytes or NEUN^+^ neurons (Figure S1). Recombination was absolutely dependent on tamoxifen induction because no YFP^+^ cells were observed anywhere in the brains of 14-month-old (P425) *Opalin–iCreER*^*T2*^*: Rosa–YFP* animals that had not received tamoxifen (not shown). This is an important control, because tamoxifen-independent recombination has been observed in *Plp1–CreER*^*T*^ mice (Traka et al., 2016) and *Mbp–CreER*^*T2*^ mice (R.B.T. and W.D.R., unpublished data), both of which express CreER at very high levels under the control of small promoter/enhancer sequences in plasmid-based transgenes.

### OLs in the Corpus Callosum Survive throughout Life

**Figure 1:**
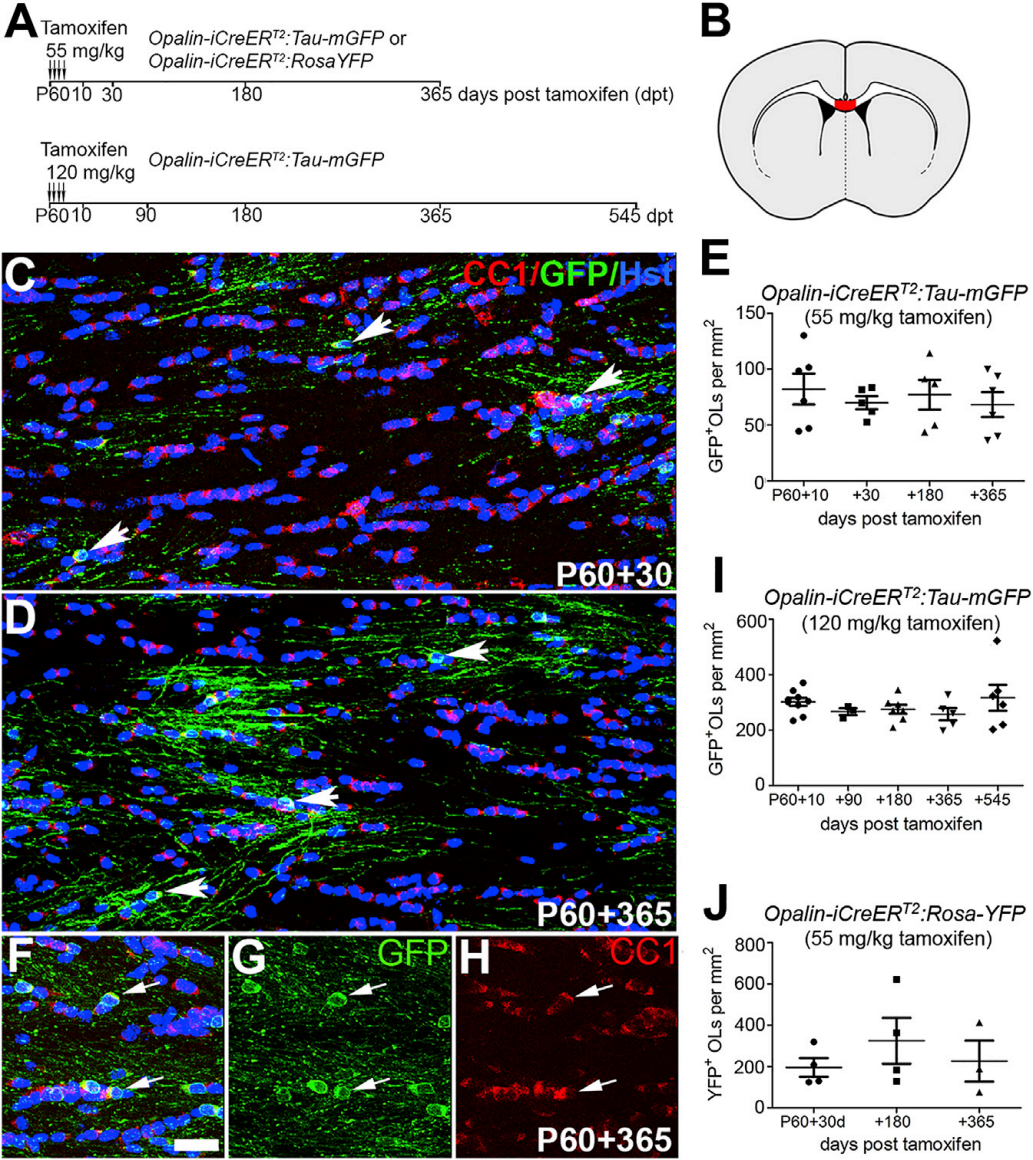
Extreme Longevity of OLs in the Corpus Callosum (A) Experimental protocol. Tamoxifen was administered to *Opalin–iCreER*^*T2*^*: Tau–mGFP* or *Opalin–iCreER*^*T2*^*: Rosa–YFP* mice starting on P60 (Experimental Procedures). Mice were killed and examined at various times post-tamoxifen as indicated. (B) Schematic of the brain showing the part of the corpus callosum (in red) within the medial limits of the lateral ventricles that was investigated in this study. (C and D) mGFP^+^, CC1^+^ OLs in *Opalin-CreER*^*T2*^*: Tau-mGFP* mice that received 55 mg/kg tamoxifen, imaged at P60+30 (C) and P60+365 (D). (E) The normalized density of mGFP^+^ OLs did not change detectably over this time frame. (F–H) mGFP^+^, CC1^+^ OLs in a mouse that received 120 mg/kg tamoxifen, imaged at P60+365. The mGFP (G) and CC1 (H) channels are merged with DAPI labelling in (F). (I) The normalized density of mGFP^+^ OLs did not change appreciably between P60+10 and P60+545. (J) Similar data were obtained using *Opalin-CreER*^*T2*^*: Rosa-YFP* mice. The normalization procedure is described in Experimental Procedures. Arrows indicate cell bodies of mGFP^+^, CC1^+^ double-positive OLs. Error bars represent SEM. Scale bar, 20 μm. See also Figures S1 and S2.

We used *Opalin–CreER*^*T2*^ to induce labeling of myelinating OLs in young adult (P60) mice in order to follow OL survival in the corpus callosum post-tamoxifen (Figures 1A and 1B). We used the *Tau–mGFP* reporter in preference to *Rosa–YFP*, because mGFP labeled the plasma membrane and revealed full cell morphology, confirming that the mGFP-labeled OLs were myelinating (Figures 1C, 1D, and 1F–1H). We confirmed that there were no mGFP^+^ cells anywhere in the forebrain, spinal cord, or optic nerves of *Opalin–CreER*^*T2*^*: Tau–mGFP* mice (P65 or P240) that did not receive tamoxifen. Following tamoxifen injection at 55 or 120 mg/kg body weight, all mGFP^+^ cells were also CC1^+^ OLs. We detected no decline in OL numbers between P60+10 and P60+365 in mice that received 55 mg/kg tamoxifen (82 ± 14 versus 68 ± 11 OLs per mm^2^; n = 6 at both time points; 1-way ANOVA, p = 0.81) (Figure 1E), or between P60+10 and P60+545 in mice that received 120 mg/kg tamoxifen (302 ± 14 versus 317 ± 47 OLs per mm^2^, n = 9, 6; 1-way ANOVA, p = 0.51) (Figure 1I). This implies that the great majority of myelinating OLs that are present in the corpus callosum at P60 are still alive and myelinating 1.5 years later.

In case the *Tau–mGFP* reporter had introduced some unexpected bias (e.g., by preferentially labeling a subset of especially long-lived OLs), we repeated these experiments using *Rosa–YFP.* As before, we could detect no loss of YFP-labeled OLs in the corpus callosum between P60+30 and P60+365 (195 ± 45 versus 225 ± 99 OLs per mm^2^, n = 4, 3 respectively; 1-way ANOVA, p = 0.56) (Figure 1J).

### OL Survival Is Region Dependent

**Figure 2:**
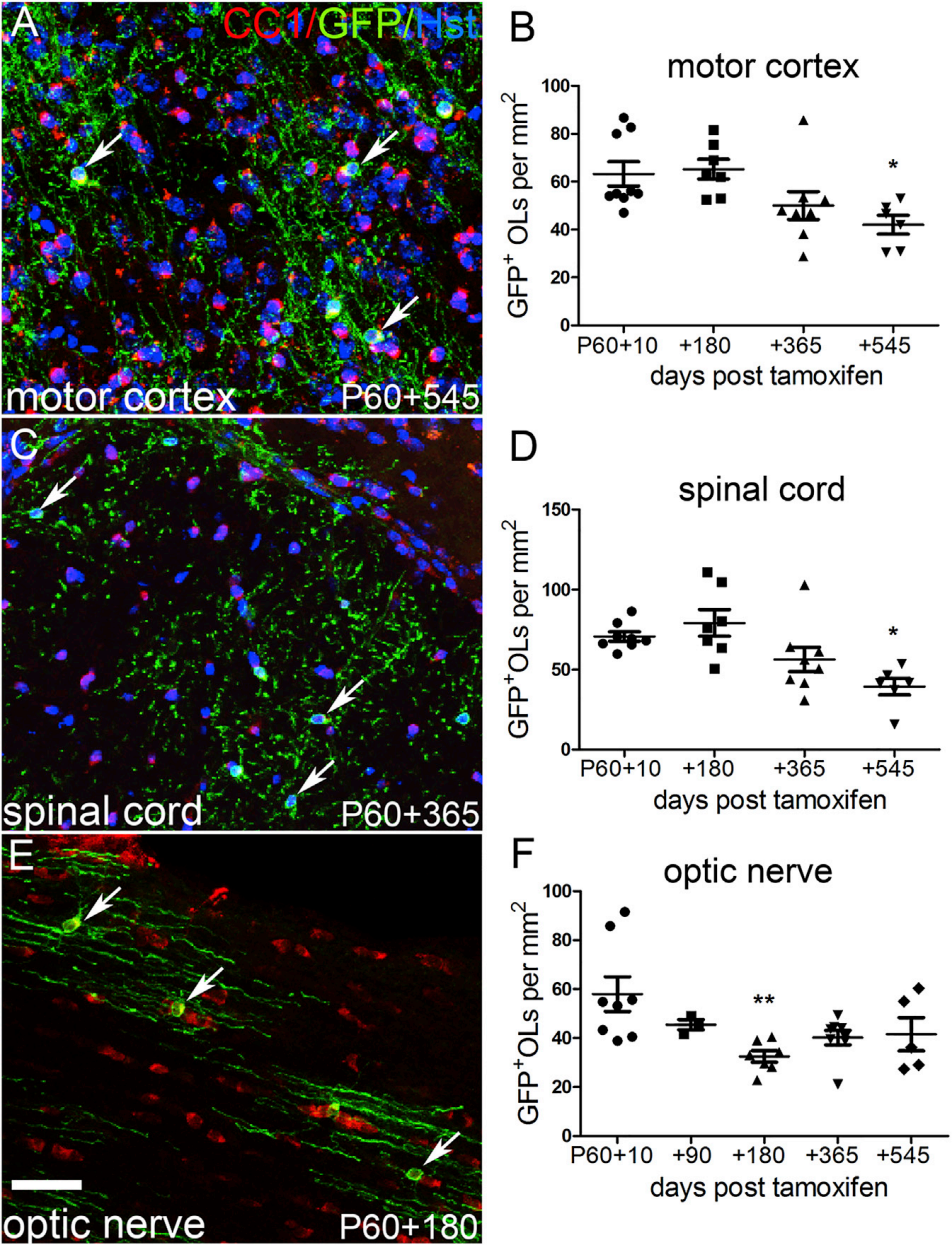
OL Survival in the Motor Cortex, Spinal Cord, and Optic Nerve *Opalin-iCreER*^*T2*^*: Tau-mGFP* mice received 120 mg/kg tamoxifen at P60 and were analyzed up to 18 months (545 days) later. (A, C, and E) mGFP^+^, CC1^+^ OLs in *Opalin-CreER*^*T2*^*: Tau-mGFP* motor cortex (A), corticospinal tract (C) or optic nerve (E), imaged at the indicated times post-tamoxifen. (B) In motor cortex, the normalized density of mGFP^+^ OLs did not change between P60+10 and P60+180 but decreased ∼30% in the following 6 months to one year (P60+545; 1-way ANOVA, p = 0.036). (D) In the corticospinal tract, the density of mGFP^+^ OLs did not change between P60+10 and P60+180, but declined during the following year (1-way ANOVA, p < 0.01). (F) In optic nerve, the density of mGFP^+^ OLs declined ∼40% between P60+10 and P60+180 (t-test, p < 0.01), after which OL density appeared to stabilize (1-way ANOVA over the whole time course, p = 0.014). Asterisks indicate values significantly different from P60+10 (^∗^p < 0.05, ^∗∗^p < 0.01, and ^∗∗∗^p < 0.001). Error bars represent SEM. Scale bar, 40 μm. See also Figures S1, S2.

We examined the motor cortex, spinal cord, and optic nerves of *Opalin–CreER*^*T2*^*: Tau–mGFP* mice that received 120 mg/kg tamoxifen starting on P60. In the motor cortex, we detected no loss of mGFP^+^ OLs between P60+10 and P60+180 (63 ± 5 versus 65 ± 4 OLs per mm^2^, n = 9, 7 respectively) (Figures 2A and 2B). However, there was a ∼33% loss of OLs between P60+10 and P60+545 (to 42 ± 4 OLs per mm^2^, n = 6, p < 0.05; 1-way ANOVA, p = 0.012). OLs were also lost over time in the corticospinal tract (CST) (Figures 2C and 2D) and optic nerve (Figures 2E and 2F). In the CST, ∼44% of OLs were lost between P60+10 and P60+545 (71 ± 3 versus 40 ± 5 OLs per mm^2^, n = 8, 6 respectively, p < 0.05; 1-way ANOVA, p = 0.002). In the optic nerve ∼45% of OLs were lost between P60+10 and P60+180 (58 ± 7 versus 32 ± 2 OLs per mm^2^, n = 8, 7 respectively, p < 0.01; 1-way ANOVA, p = 0.014).

### OL Survival after 14 Months of Age

Lasiene et al. (2009) reported that OL lineage cells (Olig2^+^) in the mouse spinal cord white matter start to increase their rate of division, estimated by bromodeoxyuridine incorporation, after 14 months of age. We imagined that the rate of OP division might increase as part of a homeostatic response to increasing death of myelinating OLs after 14 months (P425). To test this, we administered tamoxifen to *Opalin–iCreER*^*T2*^*: Tau–mGFP* mice at P425 and examined them 30 days or 180 days later. We could not detect any decrease in numbers of mGFP^+^ OLs between P425+30 and P425+180 in the corpus callosum, motor cortex, or spinal cord (Figure S2). These data are consistent with our earlier experiments in which OLs were labeled at P60 (see previous paragraph) and confirm that a large fraction of myelinating OLs present at 2 months of age survive until at least 20 months of age (P60+545 or P425+180). The actual fraction varies across the CNS; 50%–60% of OLs survive for 20 months in the corticospinal tracts and optic nerve, ∼70% in the motor cortex, and ∼90% in the corpus callosum. Over the first 6–8 months after their formation (up to P60+180), OL survival rates are 90%–100% in all regions except for the optic nerve.

### The Number of CC1^+^ OLs in the Corpus Callosum Continues to Increase after P60

**Figure 3:**
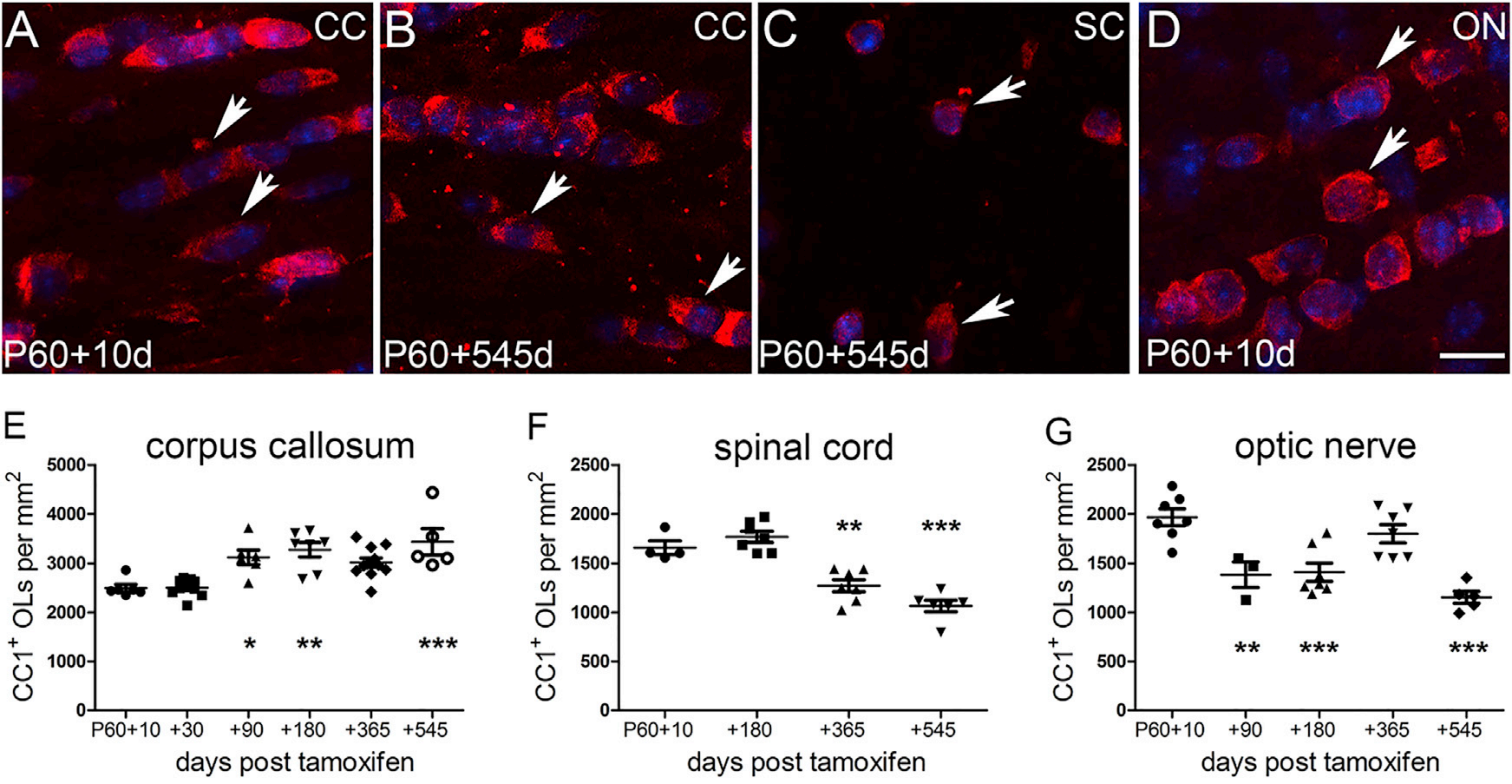
Total Number of Myelinating OLs as a Function of Age CC1^+^ mature OL cell bodies were counted in *Opalin–iCreER*^*T2*^*: Tau–mGFP* mice or wild-type mice of the same ages. (A–D) Micrographs of CC1^+^ OLs, counterstained with DAPI, in corpus callosum (A and B), corticospinal tract (C) and optic nerve (D), at the indicated times post-tamoxifen. (E) In the corpus callosum, the normalized density of CC1^+^ OLs increased between P60+10 and P60+180 and then remained approximately constant until at least P60+545 (1-way ANOVA, p < 0.0001). (F) In the spinal cord, there was a gradual decrease in the density of CC1^+^ OLs after P60+180 (1-way ANOVA, p < 0.0001) in parallel with the loss of GFP^+^ OLs (Figure 2D). (G) In the optic nerve, the density of CC1^+^ OLs decreased between P60+10 and P60+90 (1-way ANOVA, p < 0.0001) but stabilized after that, apart from a transient increase around P60+365. Asterisks indicate values significantly different from P60+10 (^∗^p < 0.05, ^∗∗^p < 0.01, and ^∗∗∗^p < 0.001). Error bars represent mean ± SEM. CC, corpus callosum; ON, optic nerve; SC, spinal cord. Scale bar, 10 μm.

Assuming that mGFP-labeled OLs in *Opalin–iCre: Tau–mGFP* mice are representative of the OL population as a whole, the absence of significant loss of mGFP^+^ OLs from the corpus callosum after P60, coupled with the fact that new OLs continue to be generated from PDGFRA^+^ OPs during young adulthood (Dimou et al., 2008, Rivers et al., 2008, Young et al., 2013), predicts that the total number of OLs in the corpus callosum should continue to increase after P60. To test this, we counted CC1^+^ differentiated OLs in the corpus callosum of our tamoxifen-treated mice at ages P60+10 and above (up to P60+545 [20 months]) (Figures 3A, 3B, and 3E). The densities of CC1^+^ OLs increased between P60+10 and P60+180 (from 2,495 ± 75 to 3,275 ± 150; p < 0.01) but remained approximately constant after that (1-way ANOVA over the full time course, p < 0.0001) (Figure 3E). These data are consistent with our previous conclusion (Young et al., 2013) that new OLs are produced in the corpus callosum for at least the first 8 months of postnatal life in mice but that their rate of production slows down with age (see Discussion).

In the corticospinal tracts (Figures 3C and 3F), unlike the corpus callosum, the density of CC1^+^ OLs followed a very similar time course to the density of mGFP^+^ OLs after P60+10 (compare with Figure 2D), confirming that mGFP-labeled OLs are representative of the general OL population; that is, the *Tau–mGFP* reporter does not introduce bias toward some subset of unusually long- or short-lived OLs.

In the optic nerve (Figures 3D and 3G), the densities of CC1^+^ and mGFP^+^ OLs also followed a similar pattern, in that both dropped in the first 180 days after P60, although the number of CC1^+^ OLs appeared to recover somewhat by 14 months of age before falling again at 20 months (compare Figures 2F and 3G). The divergence between the relative numbers of CC1^+^ and mGFP^+^ OLs at later ages might reflect the continuing production of new OLs that is known to occur in the optic nerve after P60 (Young et al., 2013), although the situation is complex, because the number of retinal ganglion cell axons in the nerve might also change with age (see Discussion).

### The Number of Internodes per OL in the Motor Cortex Is Surprisingly Stable

**Figure 4:**
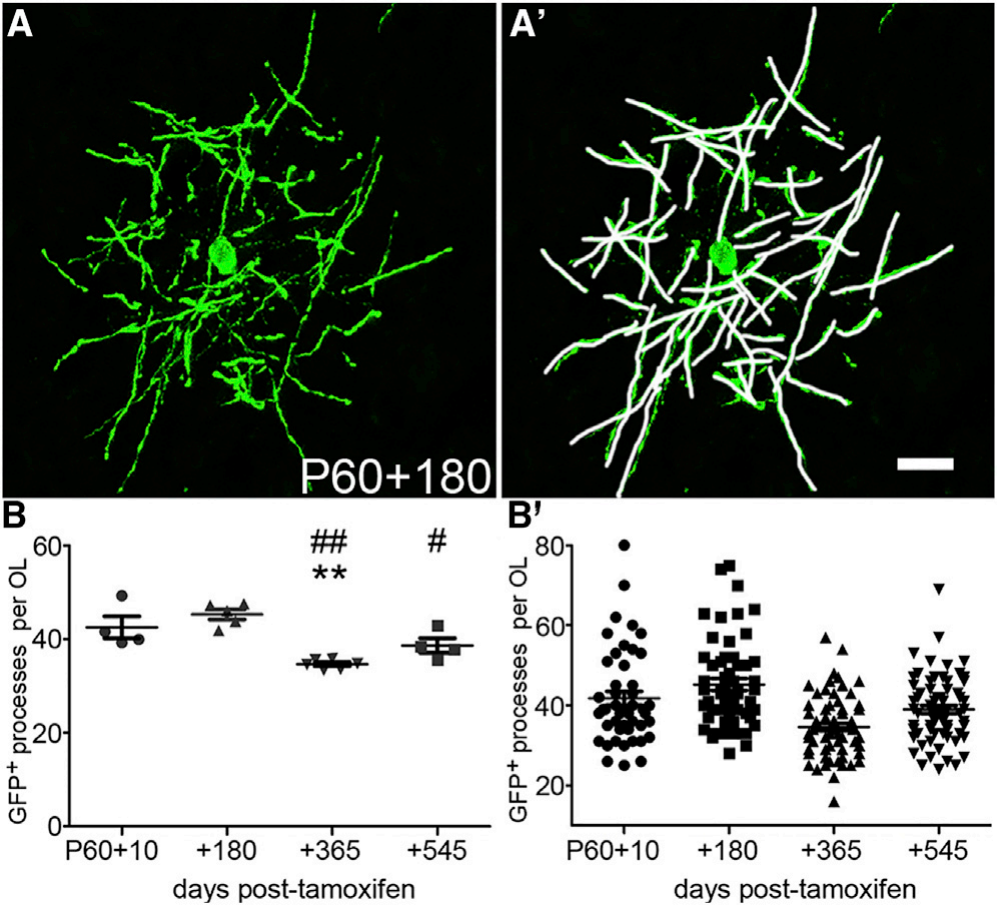
Stability of Internode Number with Age (A and A′) Individual OLs were imaged in the motor cortex of *Opalin–iCreER*^*T2*^*: Tau–mGFP* animals, and the primary cell processes/myelin internodes were marked and counted (white lines in A′). Scale bar, 20 μm. (B) There was a small but significant decrease in the number of internodes over the course of the study (1-way ANOVA, p < 0.0001). Each data point in (B) is the average from all OLs analyzed in a given mouse. Asterisks indicate values significantly different from P60+10 (^∗∗^p < 0.01), and number signs indicate values significantly different from P60+180 (^#^p < 0.05 and ^##^p < 0.01). (B′) The number of processes/internodes plotted separately for each individual OL (47 OLs at P60+10, 56 OLs at P60+180, and 66 OLs both at P60+365 and P60+545; n ≥ 4 mice at each age). Error bars represent mean ± SEM.

The *Tau-mGFP* reporter labels the external membranes of OLs, including the outer cytoplasmic loops that run along the compact myelin sheaths (Young et al., 2013). The internodes of cortical OLs formed a roughly spheroidal array centered on the cell body (Figures 4A and 4A′); they projected randomly in all directions, unlike the parallel “bundle of sticks” arrangement observed in white matter tracts. The diameter of these internode clusters was ∼150 μm (range, 120–190 μm), so they were not completely contained within a single 25-μm section (∼25% of the sphere is within the section; the fractional volume of a slice of thickness *t* through the center of a sphere of diameter *d* is {π(*d*/2)^2^ × t}/{4π(*d*/2)^3^/3} = 3*t*/2*d*, for *t* ≪ d). Nevertheless, since all cell processes originate at the cell body, we could be confident that, by selecting OLs whose cell bodies were positioned roughly at the midpoint of the section, a substantial fraction of their processes/internodes were visible over at least part of their lengths. In support of this, we counted 42 ± 3 internodes per OL at P60+10 (47 OLs examined in 4 mice; Figure 4B), which is close to a previous estimate (in 150-μm-thick sections) of ∼47 processes per OL in the frontal cortex of 8-week-old mice (Murtie et al., 2007). There was considerable individual variation among cells, ranging from ∼20 to ∼80 processes/internodes per OL at P60+10, for example (Figure 4B′). Six months later, at P60+180, the average number of processes per OL was unchanged (45 ± 2 per OL, 56 OLs examined in 5 mice). However, at 12 months and 18 months post-tamoxifen (P60+365 and P60+545), there was a significant reduction in the number of processes (35 ± 0.5 [p < 0.01] processes per cell at P60+365, 67 OLs in 6 mice and 39 ± 1.5 [p < 0.001] processes per OL at P60+545, 66 OLs in 4 mice; 1-way ANOVA, p < 0.0001) (Figure 4B). Thus, between 7% and 17% of the OL processes/internodes present at P60 were lost between 6 months and 1 year of age.

## Discussion

### Longevity of Myelinating OLs in the Forebrain

The most striking finding of our study is that >90% of myelinating OLs that are present in the corpus callosum at P60 are still alive and myelinating at 20 months of age. This corresponds to a half-life (t_1/2_) >10 years, which is substantially longer than the lifespan of the mouse (2–3 years under laboratory conditions). Thus, the great majority of myelinating OLs in mouse corpus callosum are not replaced during the animal’s lifetime.

We showed previously that new myelinating OLs continue to be generated from OPs in the mouse corpus callosum after P60 (Young et al., 2013). In that study, we labeled OPs specifically by administering tamoxifen to *Pdgfra–CreER*^*T2*^*: Rosa–YFP* mice at P60 and subsequently estimated the rate of appearance of YFP^+^, CC1^+^ OLs. At P60+42, we found that 41% ± 3% of all YFP^+^ cells were CC1^+^. That is, *N*/(*N + P*) = 0.41, where *N* is the number of YFP^+^, CC1^+^ OLs and *P* is the number of YFP^+^, CC1-negative OPs. From this, we deduce that N = 0.41/(1 − 0.41) × *p* = 0.69*P.* Given that *P* is roughly constant after P60 (∼180 OPs per mm^2^ in a 25-μm-thick section of corpus callosum) (Rivers et al., 2008, McKenzie et al., 2014), we calculate that N = 125, i.e., ∼125 new OLs per mm^2^ are formed in the 42 days after P60, or ∼3 new OLs per mm^2^ per day on average, in a 25-μm section. Young et al. (2013) also found that 69% ± 4% of YFP^+^ cells were CC1^+^ at P60+320. Psachoulia et al. (2009) found that 18% of YFP^+^ cells were PDGFRA-negative OLs at P240+100. From these data, we can calculate, as above, that the daily production rates of new myelinating OLs are on average ∼3.0 per mm^2^ per day between P60 and P102, ∼1.5 per mm^2^ per day between P60 and P380, and ∼0.40 per mm^2^ per day between P240 and P340. This illustrates that the rate of OL generation slows markedly with age, in line with the decreasing rate of OP cell division (Psachoulia et al., 2009, Young et al., 2013).

From the above, we calculate that ∼400 new OLs per mm^2^ (in a 25-μm-thick section) are generated in the corpus callosum between P60 and P380. The total number of CC1^+^ OLs increased by ∼500 OLs per mm^2^ after P60 (Figure 3), approximately matching the number of new OLs created (∼400). This simple observation indicates that most or all myelinating OLs that are generated in the corpus callosum after P60 probably survive long-term, adding to the preexisting population rather than replacing OLs that die in use. Thus, we can be confident that new callosal OLs added during the first 6–8 months of a mouse’s life, at least, are not required to replace dying OLs but rather add to the existing OL population, most likely generating new myelin sheaths on previously unmyelinated or partially myelinated axons. Up to 70% of axons in the mouse corpus callosum are still unmyelinated at 8 months of age, so there is plenty of scope for de novo myelination (Sturrock, 1980). The new myelin would be expected to modify circuit properties, contributing to experience-dependent neural plasticity.

### OL Generation and Survival in the Mouse versus Human Corpus Callosum

The average annual production rates extrapolated from the daily rates given above are ∼43% of the total OL population at ∼P81 (P60 to P60+42), ∼15% at ∼P220 (P60 to P60+320), and ∼5.5% at ∼P290 (P240 to P240+100). A recent study of OL lifetime in humans, using an environmental radiocarbon dating approach (Yeung et al., 2014), found that most OLs in the corpus callosum are formed in the first 5–10 childhood years, after which the rate of OL genesis declines to ∼0.3% of the total OL population annually. Yeung et al. (2014) assumed that the final low rate of OL genesis reflects OL “turnover” (i.e., replacement of OLs that die during adult life). However, their data do not exclude the possibility that adult-born OLs in humans might add to the preexisting OL population (as we have shown for mice) rather than replace lost cells. It is evident from our counts of total CC1^+^ OLs that the number of these cells in the mouse corpus callosum reaches a plateau before ∼8 months of age (∼20% of lifespan) compared to the first 5–10 years in human (∼10% of lifespan). Therefore, the pattern of OL generation in mouse corpus callosum is probably not fundamentally different to that of the human corpus callosum, except that the time taken for the rate of OL genesis to decline to baseline (the “developmental” period) occupies a larger fraction of a mouse’s lifespan compared to a human’s.

### Why Do OLs Survive Longer in Some Regions than in Others?

The lifetime of myelinating OLs in the optic nerve (t_1/2_ ∼2 years) contrasts with the corpus callosum (t_1/2_ >10 years) and raises the question of what controls OL survival differentially in these white matter tracts. One possibility is that there is competition among OLs for survival factors associated with or released from axons or other cells and that these factors are more limiting in the optic nerve than in the corpus callosum. A clear difference between the corpus callosum and the optic nerve is the earlier onset and greater final extent of myelination in the latter. In the rat optic nerve, ∼85% of axons are myelinated by P28 (Skoff et al., 1976), and myelination in the C57BL6/CBA mouse optic nerve follows a similar course, with practically all axons becoming myelinated by P112 (Dangata et al., 1996, Dangata and Kaufman, 1997). In contrast, ∼70% of axons in the corpus callosum remain unmyelinated, even in 8-month-old mice (Sturrock, 1980). Therefore, it is possible that there is more intense competition among OLs for factors released from unmyelinated axons in optic nerve than in corpus callosum.

Alternatively, or in addition, a subset of retinal ganglion cell (RGC) axons in the optic nerve might be lost (along with their associated OLs) during the postnatal period. Danias et al. (2003) reported a 46% loss of RGCs between 3 and 18 months of age in healthy C57BL6 mice, and Neufeld and Gachie (2003) reported a 41% loss of RGCs between 4 and 18 months in BALB/c mice. In contrast, Samuel et al. (2011) could detect no loss of RGCs in C57BL6 mice between ∼4 and ∼26 months. Nevertheless, axonal loss might account for some or all of the loss of myelinating OLs detected in our present study.

∼45% of mGFP^+^ OLs in the CST were lost between P60+10 and P60+545 (20 months). Over the same period, the number of CC1^+^ OLs declined by ∼36%. In the rat CST, ∼50% of axons and their collaterals are lost between birth and P90, without corresponding loss of neuronal cell bodies in the cortex (Gorgels et al., 1989, Stanfield, 1992). In the mouse CST, ∼68% of axons are lost between P14 and P56 (Uematsu et al., 1996). This is prior to the start of our experiments on P60, so it is unclear whether the decline in OL number that we observed in the CST might be secondary to axonal loss.

### Morphological Stability of Cortical OLs

The number of myelin internodes per OL did not change between P60+10 and P60+180 (Figure 4), although there was a small reduction (∼12%) over the following year. We cannot tell whether the very same internodes are preserved over this time or whether cortical OLs shed and reform internodes while maintaining their number approximately constant. Live imaging of developing OLs in zebrafish spinal cord showed that some OLs shed a fraction of their internodes in the first few days after their formation, but the surviving internodes were stable for at least 2–3 weeks, and no new internodes were added after the first 5 hr (Czopka et al., 2013). Therefore, it seems possible that once an OL’s arbor of internodes has been established during development, that arbor is preserved essentially unchanged for many months in the mouse, even upwards of a year. However, our analysis cannot detect dynamic changes in internode number (e.g., in response to altered neuronal activity) (Gibson et al., 2014, Hines et al., 2015, Mensch et al., 2015, Etxeberria et al., 2016), nor can we detect potential changes in internode length and/or thickness (number of wraps). It would be interesting to extend our analysis to later ages (e.g., >2 years) to determine whether loss of OLs or myelin might be a feature of extreme old age in mice, potentially contributing to age-related cognitive decline, as suspected from brain imaging studies in humans (e.g., Lu et al., 2013).

## Experimental Procedures

### Transgenic Animals

Mouse husbandry and experimentation were in accordance with UK Home Office regulations and UCL Ethics Committee guidelines, complying with the Animals (Scientific Procedures) Act 1986 of the United Kingdom and its Amendment Regulations (2012).

The *Opalin–iCreER*^*T2*^ mouse line was generated by inserting an *iCreER*^*T2*^*–lox.STOP.lox–frt.Km*^*r*^*.frt* cassette immediately downstream of the mouse *Opalin (Tmem10)* initiation codon in a BAC (RPCI24-510M10) from Source Bioscience, Nottingham, UK). *STOP* is a sequence of four simian virus 40 poly(A) addition sites. The *frt.Km*^*r*^*.frt* element was removed by expressing Flp-recombinase in the bacterial host, and the BAC was linearized using NotI prior to gel purification and pronuclear egg injection (C57BL6/CBA F1 hybrid donor females). Four founder mice were obtained, three of which expressed the transgene. The one with highest expression was retained for experiments and crossed into two reporter backgrounds: *Tau–lox.STOP.lox–mGFP–IRES–NLS.LacZ* (*Tau–mGFP)* (Hippenmeyer et al., 2007) and *Rosa26–lox.STOP.lox–eYFP* (*Rosa–YFP*) (Srinivas et al., 2001). They were maintained as homozygotes (*Rosa–YFP*) or heterozygotes (*Opalin–iCreER*^*T2*^ and *Tau–mGFP)* on a mixed C57BL6/CBA background.

### Genotyping

Mice carrying the *Opalin–iCreER*^*T2*^ transgene were identified by PCR using primers for *iCre*: 5′-GAGGGACTACCTCCTGTACC-3′ (forward) and 5′-TGCCCAGAGTCATCCTTGGC-3′ (reverse). Reporter mice were genotyped using primers for *GFP/YFP*: 5′-CCCTGAAGTTCATCTGCACCAC-3′ (forward) and 5′-TTCTCGTTGGGGTCTTTGCTC-3′ (reverse).

### Tamoxifen Administration

Tamoxifen was dissolved (10 mg/mL or 20 mg/mL) in an ethanol/ sunflower oil mixture (1:9 v/v) by sonication for ∼45 min prior to injection. It was administered by intraperitoneal injection at 55 or 120 mg per kg body weight (mg/kg) on four consecutive days, P60–P63 inclusive. They were analyzed at different times (days) after the last injection (referred to as P60+10, P60+30, etc.). Alternatively, they were injected on P425–P428 and examined on P425+30 or P425+180. To minimize variation due to potential differences in recombination rates from one series of injections to another, mice from different injection series were grouped together for examination at the same time post-injection.

### Tissue Processing and Histochemistry

Approximately equal numbers of male and female mice were analyzed. Mice were fixed by trans-cardiac perfusion with PBS at 20°C–25°C (10 mL at 10–14 mL/min) followed by 50 mL of 4% (w/v) paraformaldehyde in PBS (4% PFA) at 4°C. The brain, spinal cord, and optic nerves were dissected and post-fixed overnight by immersion in 4% PFA at 4°C. The tissues were cryo-protected with 20% (w/v) sucrose for 24–48 hr at 4°C and then embedded in optimum cutting temperature compound (Tissue-Tek) and stored at −80°C until required. Coronal cryo-sections of brain (25 μm) and spinal cord (30 μm) were transferred into PBS and immunolabeled as “floating sections.” Longitudinal sections of optic nerves (20 μm) were collected and immunolabeled directly on glass slides. Sections were blocked with 10% (v/v) fetal calf serum, 0.5% (v/v) Triton X-100 in Tris-buffered saline (pH 7.5) prior to overnight incubation with primary antibodies at 4°C. This was followed by fluorescent secondary antibody at 20°C–25°C for 1.5 hr. Cell nuclei were labeled with Hoechst 33258 (Sigma) before mounting in Dako medium (Agilent) under coverslips. Primary antibodies were mouse anti-adenomatous polyposis coli (APC) (monoclonal CC1, Millipore, 1:200 dilution), chicken anti-GFP (Aves labs, 1:1,000), mouse anti-NEUN (Millipore, 1:500), rabbit anti-PDGFRA (Cell Signaling Technologies, 1:500), and mouse anti-GFAP (Sigma, 1:500). Secondary antibodies were species-specific Alexa 488, Alexa Fluor 568, or Alexa Fluor 647 immunoglobulin G (IgG) (heavy and light chains) (Thermo Fischer Scientific, 1:1,000). To detect CC1 antigen, we used goat anti-mouse IgG2b AlexaFluor-568 (1:500).

### Quantification

We counted GFP^+^ and CC1^+^ OL cell bodies in 25-μm-thick coronal sections of the most anterior part of the corpus callosum (1.1 mm − 0.85 mm bregma), between the medial limits of the lateral ventricles, in micrographs taken with a 20× objective on a Leica SP-E confocal microscope (approximately equal numbers of males and females). We positioned the photographic field of view (a rectangle of area 0.134 mm^2^) within the region specified and expressed the number of OLs within this rectangle as OLs per mm^2^. We then measured the total area of the specified region of corpus callosum at each age using ImageJ (the area increased from 0.40 ± 0.02 mm^2^ at P60+10 to 0.46 ± 0.02 mm^2^ at P60+545) and normalized our cell densities to the area at P60+10 (for example, we multiplied the OL density at P60+545 by 46/40). This approach counters the effects of variations of tissue volume caused by age-related growth/contraction or variable shrinkage/expansion during tissue preparation. A similar approach was adopted to calculate normalized densities of GFP^+^ OLs in 25 μm sections of motor cortex, defined as the area of cortex directly dorsal to the corpus callosum as defined above. The area of this region of cortex decreased from 1.49 ± 0.07 mm^2^ to 1.21 ± 0.06 mm^2^ between P60+10 and P60+365, before increasing again to 1.96 ± 0.03 mm^2^ at P60+545. Antibody CC1 is not specific for OLs in gray matter (because it also labels many astrocytes weakly), so we did not attempt to quantify CC1^+^ OLs in the motor cortex.

In the spinal cord, we counted GFP^+^ or CC1^+^ OLs in 30-μm transverse sections of the dorsal funiculus (cervical levels C1–C2), positioning the field of view at the base of the dorsal columns (including the entire corticospinal tracts). We then measured the area of the entire dorsal funiculus (DF) (the area decreased minimally from 3.2 ± 0.08 mm^2^ at P60+10 to 2.9 ± 0.08 mm^2^ at P60+545) and normalized our cell densities to the area at P60+10 (e.g., we multiplied the measured density at P60+545 by 29/32). For the optic nerve, we counted all GFP^+^ or CC1^+^ OLs in 20-μm-thick longitudinal sections of isolated nerves and calculated OL density (OLs per mm^2^) from the measured area of the tissue section. We also measured the cross-sectional area of the intact nerve as a function of age; this did not change between P60+10 (0.073 ± 0.003 mm^2^) and P60+180 (0.071 ± 0.005 mm^2^), but it increased at P60+365 and P60+545 (0.098 ± 0.007 mm^2^ and 0.113 ± 0.003 mm^2^, respectively). The length of the nerve (5.106 ± 0.05 mm, retina to optic chiasm) did not change significantly over this period. We normalized our cell densities to the area at P60+10 (e.g., we multiplied the measured density at P60+365 by 98/73). Data are presented as mean ± SEM (n = 4–9 mice at each age).

### Statistics

Data were analyzed and plotted in GraphPad Prism 5 software using 1-way ANOVA and Bonferroni’s post hoc test for multiple comparisons, unless stated otherwise. For the older animals (tamoxifen injected on P425), a Student’s t test was used to compare means at 2 time points in 4 regions of the CNS. All data are expressed as mean ± SEM. “n” refers to the number of mice unless stated otherwise.

## Author Contributions

W.D.R. and R.B.T. conceived the project and designed the experiments. W.D.R. obtained funding. I.A.M., E.K., and M.G. contributed to the generation and characterization of transgenic mice. R.B.T. did the experiments with help from M.J. and wrote the paper with W.D.R.

## Supplemental Information

Document S1. Figures S1 and S2

Document S2. Article plus Supplemental Information
