# Supplementary material for: Remarkable Stability of Myelinating Oligodendrocytes in Mice
Source: Cell Rep. 2017 Oct 10;21(2):316–23. doi: 10.1016/j.celrep.2017.09.050 (PMC5643547; doi:10.1016/j.celrep.2017.09.050)
Supplement: Document S1. Figures S1 and S2 [file mmc1.pdf]

Cell Reports, Volume *21*

Supplemental Information

Remarkable Stability of Myelinating Oligodendrocytes in Mice

Richa B. Tripathi, Martyna Jackiewicz, Ian A. McKenzie, Eleni Kougioumtzidou, Matthew Grist, and William D. Richardson

Tripathi et al. "Remarkable stability of myelinating oligodendrocytes in mice"

SUPPLEMENTAL INFORMATION inventory:

Figure S1. Characterization of *Opalin–iCreER^{T2}* transgenic mice. Related to Figures 1 and 2.

Figure S2. Myelinating OLs persist in older mice. Related to Figures 1 and 2.

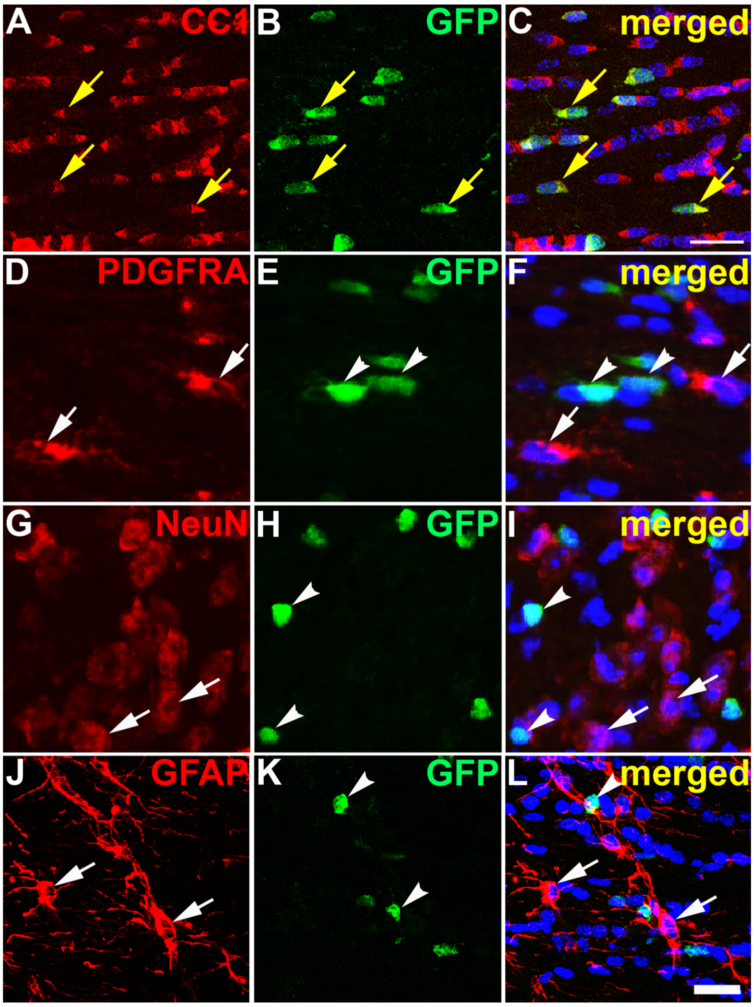

Figure S1. Characterization of *Opalin–iCreER^{T2}* transgenic mice. Related to Figures 1 and 2. (**A-C**) Tamoxifen (55 mg/kg) was injected into *Opalin–iCreER^{T2}: Rosa–YFP* mice on four consecutive days starting at P60, causing mature OLs (CC1^{+}) to become YFP-labelled. No overlap was seen between YFP and PDGFRA (OPs; **D**–**F**), NEUN (neurons; **G**–**I**) or GFAP (astrocytes; **J**–**L**). All images are of corpus callosum except G–I, which are of cerebral cortex. Cell nuclei are labelled with Hoechst 33258 (blue). ~97.5% (346/355) of YFP^{+} cells in the corpus callosum were CC1^{+} OLs, while the fraction of CC1^{+} OLs that was also YFP^{+} (i.e. the *Rosa–YFP* recombination efficiency) was 7.8% ± 1.5% (mean ± s.e.m., n=4). With the *Tau-mGFP* reporter the recombination efficiency was 2.8% ± 1.3% (n=6) after tamoxifen injection at 55 mg/kg. With 120 mg/kg tamoxifen the recombination efficiency with *Tau–mGFP* was 12.9% ± 1.6% (n=6). *White arrows* indicate cell-type specific labelling, *arrowheads* indicate GFP-labelled cells and *yellow arrows* indicate double-labelled cells. *Scale bars*, 20 µm.

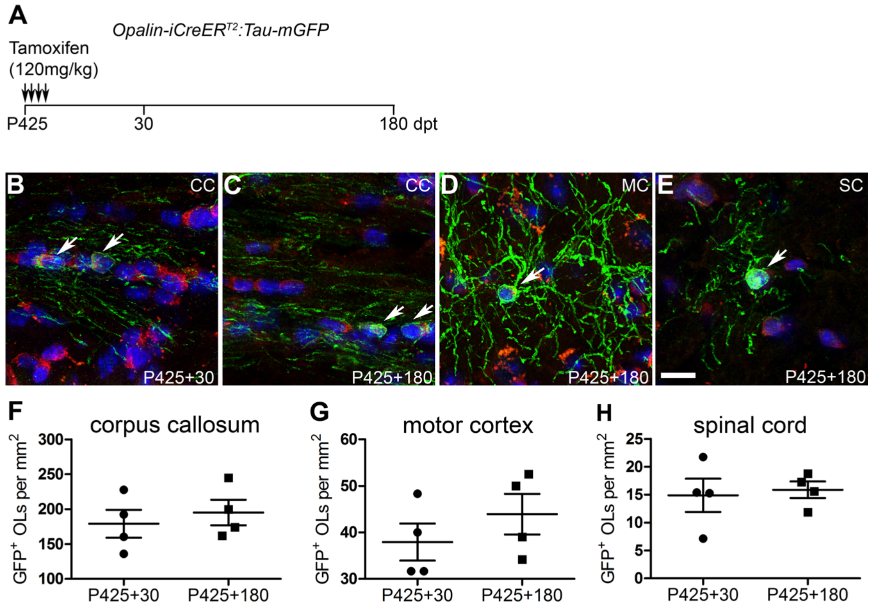

Figure S2. Myelinating OLs persist in older mice. Related to Figures 1 and 2. (**A**) Experimental protocol. Tamoxifen (120 mg/kg) was injected at P425 (14 months) on four consecutive days and mGFP^{+} OLs were counted at P425+30 and P425+180. (**B**–**E**) Sections of the regions indicated were immunolabelled with monoclonal CC1 (red), anti-GFP (green) and counter-stained with Hoechst 33258 (blue). In all regions the OL labelling efficiency at P425 was less than at P60. *CC*, corpus callosum; *MC*, motor cortex; *SC*, spinal cord. *Scale bar* 10 µm. (**F**–**H**) No significant loss of OLs was detected up to P425+180 (20 months of age) in any of the CNS regions analyzed (Student's t-test, n=4 at each age). Data are mean ± s.e.m.
